# The prostaglandin D_2_ receptor 2 pathway in asthma: a key player in airway inflammation

**DOI:** 10.1186/s12931-018-0893-x

**Published:** 2018-09-29

**Authors:** Christian Domingo, Oscar Palomares, David A. Sandham, Veit J. Erpenbeck, Pablo Altman

**Affiliations:** 1 Department of Medicine, Universitat Autònoma de Barcelona, Barcelona, Spain; 2 Pulmonary Service, Corporació Sanitària Parc Taulí, Sabadell, Barcelona, Spain; 3 Department of Biochemistry and Molecular Biology, School of Chemistry, Complutense University of Madrid, Madrid, Spain; 4 Novartis Institutes for Biomedical Research, Cambridge, MA USA; 5 Novartis Pharma AG, Basel, Switzerland; 6 Novartis Pharmaceuticals Corporation, One Health Plaza East Hanover, East Hanover, NJ 07936-1080 USA

**Keywords:** Asthma, Airway inflammation, Prostaglandin D_2_, Prostaglandin D_2_ receptor 2

## Abstract

Asthma is characterised by chronic airway inflammation, airway obstruction and hyper-responsiveness. The inflammatory cascade in asthma comprises a complex interplay of genetic factors, the airway epithelium, and dysregulation of the immune response.

Prostaglandin D_2_ (PGD_2_) is a lipid mediator, predominantly released from mast cells, but also by other immune cells such as T_H_2 cells and dendritic cells, which plays a significant role in the pathophysiology of asthma. PGD_2_ mainly exerts its biological functions via two G-protein-coupled receptors, the PGD_2_ receptor 1 (DP_1_) and 2 (DP_2_). The DP_2_ receptor is mainly expressed by the key cells involved in type 2 immune responses, including T_H_2 cells, type 2 innate lymphoid cells and eosinophils. The DP_2_ receptor pathway is a novel and important therapeutic target for asthma, because increased PGD_2_ production induces significant inflammatory cell chemotaxis and degranulation via its interaction with the DP_2_ receptor. This interaction has serious consequences in the pulmonary milieu, including the release of pro-inflammatory cytokines and harmful cationic proteases, leading to tissue remodelling, mucus production, structural damage, and compromised lung function. This review will discuss the importance of the DP_2_ receptor pathway and the current understanding of its role in asthma.

## Background

Asthma affects approximately 358 million people worldwide [1], and is characterised by chronic airway inflammation, reversible airway obstruction and hyper-responsiveness. The heterogeneous nature of this condition may cause difficulty in predicting response to treatment in a particular patient [2, 3].

Despite the availability of clinical practice guidelines and standard-of-care therapy, a large proportion of asthma patients remain symptomatic and experience poor quality-of-life [4, 5]. There is a high unmet need for novel asthma therapies, especially for patients with severe disease. Effective disease control is dependent in part by treatment adherence [6], which can be influenced by route of administration. Adherence to inhaled therapies, particularly maintenance therapies such as inhaled corticosteroids, is often poor, and is driven by the complexity of the inhaler, as well as errors during device use, such as improper actuation–inhalation coordination [7]. A clinical consequence of poor or non-adherence to inhaled therapies is increase of symptoms and eventually the occurrence of exacerbations [8]. Adherence to oral asthma treatment has been shown to be superior to that of inhaled therapies [9, 10], however oral therapy options for the management of asthma are presently quite limited. Hence, effective new oral therapies may help the management of severe or insufficiently controlled asthma [11, 12], as has been the case with the recent introduction of biological therapies via subcutaneous injection.

A treatment target with a novel mechanism of action that has gained significant interest in recent years and which has promise to be accessible by small molecule-based oral therapies, is the receptor 2 (DP_2_) of prostaglandin D_2_ (PGD_2_). This receptor is also referred to in the literature as the chemoattractant receptor homologous molecule expressed on T_H_2 cells (CRT_H_2) [13], and is expressed on the membrane surface of T_H_2 cells, type 2 innate lymphoid cells (ILC2), mast cells and eosinophils [14–16]. This review aims to discuss the current understanding of the DP_2_ receptor signalling pathway in asthma.

### Allergen-dependant and non-allergen-dependent stimulation

**Fig. 1:**
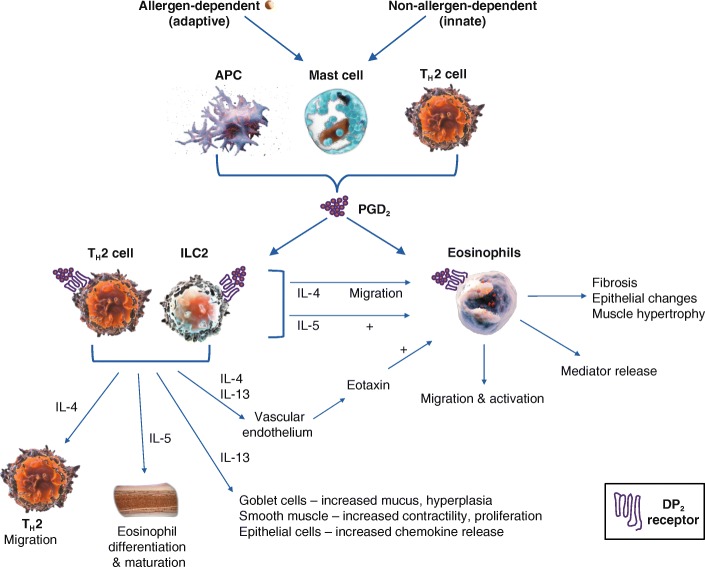
Overview of the DP_2_ receptor-mediated response of immune cells in the inflammatory pathway. Proposed schematic providing an overview of the DP_2_ receptor-mediated response of various immune cells, including mast cells, T_H_2 cells, ILC2 and eosinophils, and the subsequent effect on inflammation in the asthmatic airways through increased inflammatory cell chemotaxis and cytokine production. Abbreviations, APC: antigen presenting cell; DP_2_: prostaglandin D_2_ receptor 2; IgE: immunoglobulin E; IL: interleukin; ILC2: type 2 innate lymphoid cell; PGD_2_: prostaglandin D_2_

The inflammatory cascade in asthma comprises a complex interplay of factors. In a large proportion of patients, asthma is associated with a type 2 immune response (Type 2-high asthma) [17, 18]. Until recently, only the allergen-dependent immune pathway was considered to be an important target for asthma treatment. However, it is now clear that both the non-allergen- and allergen-dependent immune pathways are involved in the pathophysiological and immunological responses in asthma [19]. As PGD_2_, a pro-inflammatory lipid mediator, release is stimulated following both non-allergen-dependent (infections, physical stimuli or chemical stimuli) and allergen-dependent immune activation, the DP_2_ receptor pathway has relevance in both atopic and non-atopic asthma (Fig. 1) [16, 20].

### PGD_2_ release from immune cells

PGD_2_ is released following activation of the immune system, which can be either non-allergen- or allergen-dependent (Fig. 1); the non-allergen-dependent pathway comprises indirect activation of mast cells via the processing of physical agents, chemical agents or infections by antigen presenting cells, or direct activation via complement, sphingolipids and others. Through the allergen-dependent pathway, inhaled allergens trigger a cascade of events that provoke the release of PGD_2_, initiating a signalling cascade through the DP_2_ receptor in target cells (T_H_2 cells, ILC2 and eosinophils). Inhaled antigens are presented to CD4^+^ T lymphocytes by allergen presenting cells. In allergic patients, these T lymphocytes differentiate to acquire a T_H_2 cell profile, producing significant amounts of IL-4 and IL-13, which promote IgE class-switching in B lymphocytes [21–23]. Mast cells are subsequently activated upon allergen-induced cross-linking of adjacent high-affinity IgE Fc receptor (FcεRI)-bound IgE at the cell surface [24].

PGD_2_ is primarily released from mast cells through activation of hematopoietic PGD synthase, resulting in nanomolar local concentrations of the mediator [25]. Mast cells are tissue-resident cells that can be activated and degranulated in minutes [26]. They are widely distributed at mucosal surfaces and in tissues throughout the body, and play a central role in the pathophysiology of asthma, not only by mediating immunoglobulin E (IgE)-dependent allergic responses, but also in non-IgE-mediated mechanisms [27, 28]. Mast cell numbers are similarly increased in both allergic and non-allergic asthma, although response to cyclic adenosine monophosphate (cAMP) is higher in allergic than in non-allergic patients [29].

Aside from mast cells, other cell types can also produce PGD_2_ under certain conditions, including biologically meaningful quantities in T_H_2 cells [13, 30, 31]. Macrophages [32], and dendritic cells [33, 34] also produce small amounts of PGD_2_.

### PGD_2_ receptors

PGD_2_ mainly exerts its biological effect via high affinity interactions with two structurally and pharmacologically distinct receptors (the prostaglandin D_2_ receptor 1 [DP_1_] and the DP_2_ receptor) [13]. At micromolar concentrations, PGD_2_ can also stimulate the thromboxane receptor [35].

DP_1_, a 359 amino acid, ~40 kDa G-protein-coupled prostaglandin receptor, was the first PGD_2_ receptor to be identified [36, 37]. It mediates a range of effects, which are mostly non-inflammatory in nature; vasodilation, inhibition of cell migration, relaxation of smooth muscle, and eosinophil apoptosis [38].

The DP_2_ receptor is a 395 amino acid, 43 kDa G-protein-coupled prostaglandin receptor. Binding of PGD_2_ to the DP_2_ receptor on immune cells induces a myriad of pro-inflammatory downstream effects, which significantly contribute to the recruitment, activation and/or migration of T_H_2 cells, ILC2, and eosinophils, thereby fuelling the inflammatory cascade in asthma [14, 38–41]. PGD_2_ metabolites (DK-PGD_2_, Δ12PGJ2, 15-deoxy- Δ12,14PGD_2_, and deoxy- Δ12,14PGJ_2_) also activate the DP_2_ receptor [42–44].

### Cells expressing the DP_2_ receptor

The DP_2_ receptor plays a key role in the pathophysiology of asthma: it induces and amplifies the inflammatory cascade [16, 25, 45, 46]. This type of receptor can be found in many cell types, however the key cells of the DP_2_ receptor pathway include T_H_2 cells, ILC2 cells and eosinophils, suggesting a homeostatic role for this receptor (Fig. 1) [14–16, 47]. In addition, type 2 cytotoxic T (Tc2) lymphocytes were recently shown to be activated by PGD_2_ acting via the DP_2_ receptor, thus contributing to the pathogenesis of eosinophilic asthma [41].

#### Effects of the DP_2_ receptor on T_H_2 cells

PGD_2_ preferentially upregulates IL-4, IL-5 and IL-13 expression (type 2 cytokines) in T_H_2 cells in a dose-dependent manner [48] and induces T_H_2 cell migration [46] via its high affinity interaction with the DP_2_ receptor (Fig. 1).

DP_2_ receptor activation has shown a potent effect on T_H_2 cell migration in vitro, highlighting a key function of this receptor in mediating the chemotaxis of T_H_2 lymphocytes [49]. As elevated levels of circulating DP_2_^+^CD_4_^+^ T cells is a hallmark feature of severe asthma [50], this provides a DP_2_ receptor-rich environment upon which already increased levels of PGD_2_ levels may act, further perpetuating the inflammatory cascade.

#### Effects of the DP_2_ receptor on ILC2 cells

ILC2 is a cell type that may link the non-allergen- and allergen-dependent responses in asthma. ILC2 cell activation is triggered by inflammatory mediators released from epithelial and immune cells (e.g. IL-33 and PGD_2_), and is associated with increased production of type 2 cytokines [51]. Thus, ILC2 cells facilitate a T_H_2 immune response that can be independent of the allergen [52].

Secretion of IL-4, IL-5 and IL-13 from ILC2 cells is increased in response to DP_2_ receptor stimulation in a dose-dependent manner [16].

In response to IL-33, ILC2 cell activation was initially reported to produce high levels of IL-5 and IL-13 in vitro, but very low levels of IL-4. Interestingly, recent studies have shown that when their DP_2_ receptor is stimulated, ILC2 cells produce higher levels of IL-4 [53].

Meanwhile, DP_2_ stimulation alone remarkably increases ILC2 cell migration, which is 4.75-fold greater than that of IL-33 [16].

#### Effects of the DP_2_ receptor on eosinophils

Eosinophils are involved in airway hyper-responsiveness, mucus hypersecretion, tissue damage and airway remodelling in asthma. Eosinophil activation is also associated with increased cytokine production, which has various downstream immunomodulatory effects [54]. DP_2_ receptor activation at the eosinophil surface facilitates the trans-endothelial migration and influx of eosinophils, increases eosinophil degranulation and induces eosinophil shape change [40, 55, 56]. Eosinophil shape change in response to DP_2_ activation [57] is similar to that visualised previously with eotaxin stimulation [58].

Eosinophil influx and activation can cause detrimental effects on the epithelial lining of the lungs of asthma patients. This happens through degranulation and release of harmful mediators such as eosinophil cationic protein, eosinophil peroxidase, eosinophil protein X and cytotoxic major basic protein [19, 59, 60]. Additionally, eosinophils release transforming growth factor (TGF)-ß which induces apoptotic effects upon airway epithelial cells, contributing to airway tissue denudation. Moreover, eosinophils enhance airway smooth muscle cell proliferation, further contributing to structural remodelling of the pulmonary architecture [61]. Charcot-Leyden crystals, a product of activated eosinophils, are detectable in expectorated sputum samples from asthma patients [62]. These crystals are largely comprised of the toxic enzyme lysophospholipase (also known as phospholipase B), and may contribute to eosinophil-driven tissue denudation in the lungs [63].

As mentioned previously, in addition to the direct effects, DP_2_ receptor activation also has indirect effects on eosinophils by inducing the release of IL-4, IL-5 and IL-13 from T_H_2 cells and ILC2, which affect eosinophil maturation, apoptosis and migration to the lungs.

### Effects of DP_2_-mediated cytokine release

DP_2_ receptor activation increases release of cytokines from ILC2 and T_H_2 cells. These cytokines cause some of the characteristic features of asthma, including airway inflammation, IgE production, mucus metaplasia, airway hyper-reactivity, smooth muscle remodelling and eosinophilia [52, 64]. We will review the effects of the key cytokines released:

- IL-4 enhances the migration of eosinophils, which is a key step in the inflammatory cascade. To do this, in synergy with tumour necrosis factor (TNF)-α, IL-4 increases the expression of vascular cell adhesion molecule-1 (VCAM-1) and P selectin on the surface of the vascular endothelium, which facilitates the trans-endothelial passage of eosinophils from the bloodstream into the lung parenchyma [19, 65]. Meanwhile, IL-4 also stimulates the release of eotaxin, a potent and selective eosinophil chemoattractant, from the vascular endothelium (Fig. 1). Eotaxin facilitates eosinophil migration [66, 67]. Differentiation and proliferation of T_H_2 cells is also promoted by IL-4 [39].
- IL-5 is directly involved in the differentiation and maturation of eosinophils in the bone marrow, eosinophil chemotaxis to sites of inflammation, and local eosinophilopoiesis [68, 69]. It also inhibits eosinophil apoptosis, leading to the accumulation of these cells at sites of inflammation, which in turn perpetuates and prolongs the inflammatory cycle [70].
- IL-13 is known to induce goblet cell hyperplasia, mucus production, and airway hyper-responsiveness, leading to airway inflammation and tissue remodelling [39, 64]. Furthermore, IL-4 and IL-13 released from T_H_2 and ILC2 in response to DP_2_ receptor activation promote immunoglobulin class switching from IgM to IgE antibodies in B cells and plasma cells, which leads to further mast cell recruitment, activation and PGD_2_ release at sites of inflammation [16, 20, 71, 72]. It also contributes to the release of eotaxin (together with IL-4), which as mentioned above, facilitates eosinophil migration.
- Levels of other pro-inflammatory cytokines are also increased upon activation of DP_2_ receptors, including IL-8, IL-9 and granulocyte–macrophage colony-stimulating factor, which may additionally contribute to excessive immune cell chemotaxis, associated proteases and enhanced airway inflammation in asthma [16].

Results from phase II clinical studies suggest that blocking the activation of the DP_2_ receptor pathway with DP_2_ receptor antagonists reduces the symptoms associated with asthma, improves pulmonary function and inhibits eosinophil shape change, while showing indirect signs (sputum eosinophil reduction) of the potential to decrease the number of exacerbations experienced by severe asthma patients [73–80].

### Further evidence for DP_2_ receptor pathway importance in asthma

PGD_2_ levels are increased in asthma, with increased levels in patients with severe disease [27, 81], and in response to allergen challenge [82, 83]. The number of DP_2_ receptor-positive cells within the submucosal tissue is also significantly higher in patients with severe asthma compared with healthy controls [84]. Interestingly, an association between a single nucleotide polymorphism in the DP_2_ receptor (rs533116) and allergic asthma has also been reported [85].

PGD_2_ protein and DP_2_ receptor expression levels in bronchoalveolar lavage fluid (BALF) from severe asthmatic patients were shown to be significantly higher than from healthy controls or patients with mild or moderate asthma [27, 81]. Interestingly, Murray et al. [82] demonstrated a 150-fold increase in PGD_2_ levels in BALF from asthma patients within nine minutes of local antigen *(Dermatophagoides pteronyssinus)* challenge, demonstrating that allergen-induced PGD_2_ release is an early and rapid event. Furthermore, a study by Wenzel and colleagues showed that allergen challenge in atopic asthma patients induced a significant increase in BALF PGD_2_ levels compared with atopic patients without asthma [83].

Of significant interest is the sustained activity of PGD_2_-derived metabolites despite extensive and rapid PGD_2_ metabolism. The PGD_2_-derived metabolites PGJ_2_ and Δ^12^-PGJ_2_, are themselves known to be potent DP_2_ receptor agonists, thereby demonstrating the sustained and prolonged activity of the DP_2_ receptor via the metabolites of PGD_2_ [45]. Despite the short half-life of PGD_2_ in plasma (~30 min), its biological activity towards the DP_2_ receptor is maintained through the formation of these metabolites, which are more stable than the parent compound, highlighting their potential role in perpetuating the inflammatory cascade [45].

Blockage of PGD_2_ via DP_2_ receptor antagonism inhibits inflammatory cell chemotaxis and also reduces type 2 pro-inflammatory cytokine production, which provides further evidence of the vital role played by PGD_2_ and its interaction with the DP_2_ receptor in asthma [46]. Of note, DP_2_ receptor antagonism has also been shown to decrease airway smooth muscle cell mass and chemotaxis of these cells towards PGD_2_ [86, 87].

### Role of the DP_2_ receptor pathway in virus-induced asthma

Viruses, such as rhinovirus (RV), influenza A, and respiratory syncytial virus (RSV), are a major cause of asthma exacerbations and can activate the DP_2_ receptor pathway [88]. These respiratory viruses produce double-stranded RNA (dsRNA) during replication, which activates the non-allergen-dependent immune response and results in increased chemokine synthesis from airway epithelial and innate immune cells [88, 89]. A recent study also suggests the involvement of the DP_2_ receptor pathway in augmenting virus-mediated airway eosinophilic inflammation [88]. It shows that DP_2_ receptor stimulation followed by eosinophil recruitment into the airways is a major pathogenic factor in the dsRNA-induced enhancement of airway inflammation and bronchial hyper-responsiveness [88].

PGD_2_ levels have also been found to be increased after viral challenge in asthma patients, which may act synergistically with IL-33 to further drive type 2 cytokine production [90, 91]. The role of PGD_2_ in RV16-induced asthma exacerbations was recently investigated in atopic asthma patients [91]. In this study, baseline PGD_2_ levels were higher in asthmatic patients versus healthy controls. Furthermore, RV16 infection induced a greater PGD_2_ increase in asthmatic patients compared with the healthy participants. The largest RV16-mediated PGD_2_ increase was observed in those with severe and poorly-controlled asthma, suggesting a potential role for PGD_2_ in driving asthma exacerbations [91].

Polyinosinic:polycytidylic acid (poly I:C) is an immunostimulant; it is structurally similar to double-stranded RNA, which is present in some viruses and is a “natural” stimulant of toll-like receptor 3 (TLR3), which is expressed in the membrane of B-cells, macrophages and dendritic cells. Thus, poly I:C can be considered a synthetic analogue of double-stranded RNA and can simulate viral infections. Early evidence from poly I:C murine asthma models suggests that a selective DP_2_ receptor antagonist may dose-dependently block the aforementioned virus-induced T2 response, and may help to reduce the inflammation caused by virus-mediated asthma exacerbations [92].

## Conclusions

The DP_2_ receptor pathway is known to play a key role in the pathophysiology of asthma via induction and amplification of the inflammatory cascade by exerting direct effects on immune cells, including T_H_2 cella, ILC2 and eosinophils [16, 46, 55]. IL-4, IL-5 and IL-13 release from DP_2_ receptor-activated immune cells can have significant effects on immune cell influx, degranulation, tissue remodelling and mucus production in the airways, leading to structural damage, fibrosis and reduced pulmonary function [64]. Additionally, the effect of DP_2_ receptor activation on eosinophil activation and migration leads to tissue damage, through release of harmful cationic proteins and enhanced proliferation of airway smooth muscle cells [93].

This review highlights the important pro-inflammatory role of the DP_2_ receptor pathway in asthma. Furthermore, multiple DP_2_ receptor antagonists are currently under clinical investigation [73–75, 77–80], for asthma therapies. Indeed, in a 12-week study in patients with allergic asthma that was uncontrolled by low-dose ICS, the oral DP_2_ receptor antagonist fevipiprant (150 mg once daily or 75 mg twice daily) produced significant improvements in pre-dose FEV_1_ compared with placebo [73]. Further, in patients with moderate to severe eosinophilic asthma, fevipiprant significantly reduced mean sputum eosinophil percentage compared with placebo [80]. Initial positive findings have also been reported with timapiprant (OC00459) [78], BI 671800 [77], setipiprant [94] , MK-1029 and ADC-3680 [95] , but not with AZD1981 [75]. Hence, the clinical outcomes of larger, phase III clinical studies involving DP_2_ receptor antagonists are eagerly awaited.

## Abbreviations

DP_1_: Prostaglandin D_2_ receptor 1
DP_2_: Prostaglandin D_2_ receptor 2
IgE: Immunoglobulin E
IL: Interleukin
ILC2: Type 2 innate lymphoid cell
PGD_2_: Prostaglandin D_2_
Tc2: Type 2 cytotoxic T cell
TGF-β: Transforming growth factor-β
TNF-α: Tumour necrosis factor-α
VCAM-1: Vascular cell adhesion molecule-1

## References

[1] GBD Chronic Respiratory Disease Collaborators (2017). Global, regional, and national deaths, prevalence, disability-adjusted life years, and years lived with disability for chronic obstructive pulmonary disease and asthma, 1990-2015: a systematic analysis for the Global Burden of Disease Study 2015. Lancet Respir Med.

[2] Boyman O, Kaegi C, Akdis M, Bavbek S, Bossios A, Chatzipetrou A, Eiwegger T, Firinu D, Harr T, Knol E (2015). EAACI IG Biologicals task force paper on the use of biologic agents in allergic disorders. Allergy.

[3] Palomares Óscar, Sánchez-Ramón Silvia, Dávila Ignacio, Prieto Luis, Pérez de Llano Luis, Lleonart Marta, Domingo Christian, Nieto Antonio (2017). dIvergEnt: How IgE Axis Contributes to the Continuum of Allergic Asthma and Anti-IgE Therapies. International Journal of Molecular Sciences.

[4] Hetherington KJ, Heaney LG (2015). Drug therapies in severe asthma - the era of stratified medicine. Clin Med (Lond).

[5] Price D, Fletcher M, van der Molen T (2014). Asthma control and management in 8,000 European patients: the REcognise Asthma and LInk to Symptoms and Experience (REALISE) survey. NPJ Prim Care Respir Med.

[6] Eakin MN, Rand CS (2012). Improving patient adherence with asthma self-management practices: what works?. Ann Allergy Asthma Immunol.

[7] Price D, Bosnic-Anticevich S, Briggs A, Chrystyn H, Rand C, Scheuch G, Bousquet J (2013). Inhaler competence in asthma: common errors, barriers to use and recommended solutions. Respir Med.

[8] Williams LK, Peterson EL, Wells K, Ahmedani BK, Kumar R, Burchard EG, Chowdhry VK, Favro D, Lanfear DE, Pladevall M (2011). Quantifying the proportion of severe asthma exacerbations attributable to inhaled corticosteroid nonadherence. J Allergy Clin Immunol.

[9] Jones C, Santanello NC, Boccuzzi SJ, Wogen J, Strub P, Nelsen LM (2003). Adherence to prescribed treatment for asthma: evidence from pharmacy benefits data. J Asthma.

[10] Rand C, Bilderback A, Schiller K, Edelman JM, Hustad CM, Zeiger RS, Group MSR (2007). Adherence with montelukast or fluticasone in a long-term clinical trial: results from the mild asthma montelukast versus inhaled corticosteroid trial. J Allergy Clin Immunol.

[11] Barnes PJ (2004). New drugs for asthma. Nat Rev Drug Discov.

[12] Barnes PJ (2010). New therapies for asthma: is there any progress?. Trends Pharmacol Sci.

[13] Pettipher R (2008). The roles of the prostaglandin D(2) receptors DP(1) and CRTH2 in promoting allergic responses. Br J Pharmacol.

[14] Hirai H, Tanaka K, Yoshie O, Ogawa K, Kenmotsu K, Takamori Y, Ichimasa M, Sugamura K, Nakamura M, Takano S, Nagata K (2001). Prostaglandin D2 selectively induces chemotaxis in T helper type 2 cells, eosinophils, and basophils via seven-transmembrane receptor CRTH2. J Exp Med.

[15] Nagata K, Hirai H, Tanaka K, Ogawa K, Aso T, Sugamura K, Nakamura M, Takano S (1999). CRTH2, an orphan receptor of T-helper-2-cells, is expressed on basophils and eosinophils and responds to mast cell-derived factor(s). FEBS Lett.

[16] Xue L, Salimi M, Panse I, Mjosberg JM, McKenzie AN, Spits H, Klenerman P, Ogg G (2014). Prostaglandin D2 activates group 2 innate lymphoid cells through chemoattractant receptor-homologous molecule expressed on TH2 cells. J Allergy Clin Immunol.

[17] Palomares O, Akdis CA. Chapter 28 - Immunology of the Asthmatic Immune Response. In: Leung D, Szefler S, Bonilla F, Akdis CA, Sampson H, editors. Pediatric Allergy: Principles and Practice, 3rd Edition. London: Elsevier; 2015. p. 250–61.

[18] Palomares O, Akdis M, Martin-Fontecha M, Akdis CA (2017). Mechanisms of immune regulation in allergic diseases: the role of regulatory T and B cells. Immunol Rev.

[19] Domingo C (2017). Overlapping Effects of New Monoclonal Antibodies for Severe Asthma. Drugs.

[20] Townley RG, Agrawal S (2012). CRTH2 antagonists in the treatment of allergic responses involving TH2 cells, basophils, and eosinophils. Ann Allergy Asthma Immunol.

[21] Domingo C, Pacheco A, Hinojosa M, Bosque M (2007). The relevance of IgE in the pathogenesis of allergy: the effect of an anti-IgE drug in asthma and other diseases. Recent Pat Inflamm Allergy Drug Discov.

[22] Domingo C (2014). Omalizumab for severe asthma: efficacy beyond the atopic patient?. Drugs.

[23] Peinhaupt M, Sturm EM, Heinemann A (2017). Prostaglandins and Their Receptors in Eosinophil Function and As Therapeutic Targets. Front Med (Lausanne).

[24] Brightling CE, Bradding P, Pavord ID, Wardlaw AJ (2003). New insights into the role of the mast cell in asthma. Clin Exp Allergy.

[25] Pettipher R, Hansel TT, Armer R (2007). Antagonism of the prostaglandin D2 receptors DP1 and CRTH2 as an approach to treat allergic diseases. Nat Rev Drug Discov.

[26] Matsuda K, Piliponsky AM, Iikura M, Nakae S, Wang EW, Dutta SM, Kawakami T, Tsai M, Galli SJ (2005). Monomeric IgE enhances human mast cell chemokine production: IL-4 augments and dexamethasone suppresses the response. J Allergy Clin Immunol.

[27] Balzar S, Fajt ML, Comhair SA, Erzurum SC, Bleecker E, Busse WW, Castro M, Gaston B, Israel E, Schwartz LB (2011). Mast cell phenotype, location, and activation in severe asthma. Data from the Severe Asthma Research Program. Am J Respir Crit Care Med.

[28] Amin K (2012). The role of mast cells in allergic inflammation. Respir Med.

[29] Ludviksdottir D, Janson C, Bjornsson E, Stalenheim G, Boman G, Hedenstrom H, Venge P, Gudbjornsson B, Valtysdottir S (2000). Different airway responsiveness profiles in atopic asthma, nonatopic asthma, and Sjogren’s syndrome. Allergy.

[30] Vinall SL, Townsend ER, Pettipher R (2007). A paracrine role for chemoattractant receptor-homologous molecule expressed on T helper type 2 cells (CRTH2) in mediating chemotactic activation of CRTH2+ CD4+ T helper type 2 lymphocytes. Immunology.

[31] Tanaka K, Ogawa K, Sugamura K, Nakamura M, Takano S, Nagata K (2000). Cutting edge: differential production of prostaglandin D2 by human helper T cell subsets. J Immunol.

[32] Tajima T, Murata T, Aritake K, Urade Y, Hirai H, Nakamura M, Ozaki H, Hori M (2008). Lipopolysaccharide induces macrophage migration via prostaglandin D(2) and prostaglandin E(2). J Pharmacol Exp Ther.

[33] Urade Y, Ujihara M, Horiguchi Y, Ikai K, Hayaishi O (1989). The major source of endogenous prostaglandin D2 production is likely antigen-presenting cells. Localization of glutathione-requiring prostaglandin D synthetase in histiocytes, dendritic, and Kupffer cells in various rat tissues. J Immunol.

[34] Shimura C, Satoh T, Igawa K, Aritake K, Urade Y, Nakamura M, Yokozeki H (2010). Dendritic cells express hematopoietic prostaglandin D synthase and function as a source of prostaglandin D2 in the skin. Am J Pathol.

[35] Coleman RA, Sheldrick RL (1989). Prostanoid-induced contraction of human bronchial smooth muscle is mediated by TP-receptors. Br J Pharmacol.

[36] Hirata M, Kakizuka A, Aizawa M, Ushikubi F, Narumiya S (1994). Molecular characterization of a mouse prostaglandin D receptor and functional expression of the cloned gene. Proc Natl Acad Sci U S A.

[37] Boie Y, Sawyer N, Slipetz DM, Metters KM, Abramovitz M (1995). Molecular cloning and characterization of the human prostanoid DP receptor. J Biol Chem.

[38] Kupczyk M, Kuna P (2017). Targeting the PGD2/CRTH2/DP1 Signaling Pathway in Asthma and Allergic Disease: Current Status and Future Perspectives. Drugs.

[39] Arima M, Fukuda T (2011). Prostaglandin D(2) and T(H)2 inflammation in the pathogenesis of bronchial asthma. Korean J Intern Med.

[40] Sykes DA, Bradley ME, Riddy DM, Willard E, Reilly J, Miah A, Bauer C, Watson SJ, Sandham DA, Dubois G, Charlton SJ (2016). Fevipiprant (QAW039), a Slowly Dissociating CRTh2 Antagonist with the Potential for Improved Clinical Efficacy. Mol Pharmacol.

[41] Xue L, Stöger L, Marchi E, Liu W, Go S, Kurioka A, Leng T, Willberg C, Salimi M, Shrimanker R (2017). Interaction of Type 2 cytotoxic T lymphocytes and mast cell lipid mediators contributes to pathogenesis of eosinophilic asthma. Am J Respir Crit Care Med.

[42] Gazi L, Gyles S, Rose J, Lees S, Allan C, Xue L, Jassal R, Speight G, Gamble V, Pettipher R (2005). Delta12-prostaglandin D2 is a potent and selective CRTH2 receptor agonist and causes activation of human eosinophils and Th2 lymphocytes. Prostaglandins Other Lipid Mediat.

[43] Monneret G, Li H, Vasilescu J, Rokach J, Powell WS (2002). 15-Deoxy-delta 12,14-prostaglandins D2 and J2 are potent activators of human eosinophils. J Immunol.

[44] Sawyer N, Cauchon E, Chateauneuf A, Cruz RP, Nicholson DW, Metters KM, O'Neill GP, Gervais FG (2002). Molecular pharmacology of the human prostaglandin D2 receptor, CRTH2. Br J Pharmacol.

[45] Schuligoi R, Schmidt R, Geisslinger G, Kollroser M, Peskar BA, Heinemann A (2007). PGD2 metabolism in plasma: kinetics and relationship with bioactivity on DP1 and CRTH2 receptors. Biochem Pharmacol.

[46] Xue L, Barrow A, Fleming VM, Hunter MG, Ogg G, Klenerman P, Pettipher R (2012). Leukotriene E4 activates human Th2 cells for exaggerated proinflammatory cytokine production in response to prostaglandin D2. J Immunol.

[47] Chang JE, Doherty TA, Baum R, Broide D (2014). Prostaglandin D2 regulates human type 2 innate lymphoid cell chemotaxis. J Allergy Clin Immunol.

[48] Xue L, Gyles SL, Wettey FR, Gazi L, Townsend E, Hunter MG, Pettipher R (2005). Prostaglandin D2 causes preferential induction of proinflammatory Th2 cytokine production through an action on chemoattractant receptor-like molecule expressed on Th2 cells. J Immunol.

[49] Gyles SL, Xue L, Townsend ER, Wettey F, Pettipher R (2006). A dominant role for chemoattractant receptor-homologous molecule expressed on T helper type 2 (Th2) cells (CRTH2) in mediating chemotaxis of CRTH2+ CD4+ Th2 lymphocytes in response to mast cell supernatants. Immunology.

[50] Palikhe NS, Laratta C, Nahirney D, Vethanayagam D, Bhutani M, Vliagoftis H, Cameron L (2016). Elevated levels of circulating CD4(+) CRTh2(+) T cells characterize severe asthma. Clin Exp Allergy.

[51] Chen R, Smith SG, Salter B, El-Gammal A, Oliveria JP, Obminski C, Watson R, O'Byrne PM, Gauvreau GM, Sehmi R (2017). Allergen-induced Increases in Sputum Levels of Group 2 Innate Lymphoid Cells in Subjects with Asthma. Am J Respir Crit Care Med.

[52] Karta MR, Broide DH, Doherty TA (2016). Insights into Group 2 Innate Lymphoid Cells in Human Airway Disease. Curr Allergy Asthma Rep.

[53] Lund S, Walford HH, Doherty TA (2013). Type 2 Innate Lymphoid Cells in Allergic Disease. Curr Immunol Rev.

[54] McBrien CN, Menzies-Gow A (2017). The Biology of Eosinophils and Their Role in Asthma. Front Med (Lausanne).

[55] Gervais FG, Cruz RP, Chateauneuf A, Gale S, Sawyer N, Nantel F, Metters KM, O'Neill GP (2001). Selective modulation of chemokinesis, degranulation, and apoptosis in eosinophils through the PGD2 receptors CRTH2 and DP. J Allergy Clin Immunol.

[56] Sandham DA, Barker L, Brown L, Brown Z, Budd D, Charlton SJ, Chatterjee D, Cox B, Dubois G, Duggan N (2017). Discovery of Fevipiprant (NVP-QAW039), a Potent and Selective DP2 Receptor Antagonist for Treatment of Asthma. ACS Med Chem Lett.

[57] Royer JF, Schratl P, Carrillo JJ, Jupp R, Barker J, Weyman-Jones C, Beri R, Sargent C, Schmidt JA, Lang-Loidolt D, Heinemann A (2008). A novel antagonist of prostaglandin D2 blocks the locomotion of eosinophils and basophils. Eur J Clin Invest.

[58] Willetts L, Ochkur SI, Jacobsen EA, Lee JJ, Lacy P, Walsh GM (2014). Eosinophil Shape Change and Secretion. Eosinophils Methods in Molecular Biology (Methods and Protocols) Volume 1178.

[59] Frigas E, Motojima S, Gleich GJ (1991). The eosinophilic injury to the mucosa of the airways in the pathogenesis of bronchial asthma. Eur Respir J Suppl.

[60] Carr TF, Berdnikovs S, Simon HU, Bochner BS, Rosenwasser LJ (2016). Eosinophilic bioactivities in severe asthma. World Allergy Organ J.

[61] Halwani R, Vazquez-Tello A, Sumi Y, Pureza MA, Bahammam A, Al-Jahdali H, Soussi-Gounni A, Mahboub B, Al-Muhsen S, Hamid Q (2013). Eosinophils induce airway smooth muscle cell proliferation. J Clin Immunol.

[62] Dor PJ, Ackerman SJ, Gleich GJ (1984). Charcot-Leyden crystal protein and eosinophil granule major basic protein in sputum of patients with respiratory diseases. Am Rev Respir Dis.

[63] Weller PF, Goetzl EJ, Austen KF (1980). Identification of human eosinophil lysophospholipase as the constituent of Charcot-Leyden crystals. Proc Natl Acad Sci U S A.

[64] Farne H, Jackson DJ, Johnston SL (2016). Are emerging PGD2 antagonists a promising therapy class for treating asthma?. Expert Opin Emerg Drugs.

[65] Patel KD (1998). Eosinophil tethering to interleukin-4-activated endothelial cells requires both P-selectin and vascular cell adhesion molecule-1. Blood.

[66] Conroy DM, Williams TJ (2001). Eotaxin and the attraction of eosinophils to the asthmatic lung. Respir Res.

[67] Moore PE, Church TL, Chism DD, Panettieri RA, Shore SA (2002). IL-13 and IL-4 cause eotaxin release in human airway smooth muscle cells: a role for ERK. Am J Physiol Lung Cell Mol Physiol.

[68] Aceves SA, Ackerman SJ (2009). Relationships Between Eosinophilic Inflammation, Tissue Remodeling and Fibrosis in Eosinophilic Esophagitis. Immunology Allergy Clin North Am.

[69] Kouro T, Takatsu K (2009). IL-5- and eosinophil-mediated inflammation: from discovery to therapy. Int Immunol.

[70] Molfino NA, Gossage D, Kolbeck R, Parker JM, Geba GP (2012). Molecular and clinical rationale for therapeutic targeting of interleukin-5 and its receptor. Clin Exp Allergy.

[71] Punnonen J, Aversa G, Cocks BG, McKenzie AN, Menon S, Zurawski G, de Waal MR, de Vries JE (1993). Interleukin 13 induces interleukin 4-independent IgG4 and IgE synthesis and CD23 expression by human B cells. Proc Natl Acad Sci U S A.

[72] Vatrella A, Fabozzi I, Calabrese C, Maselli R, Pelaia G (2014). Dupilumab: a novel treatment for asthma. J Asthma Allergy.

[73] Bateman Eric D., Guerreros Alfredo G., Brockhaus Florian, Holzhauer Björn, Pethe Abhijit, Kay Richard A., Townley Robert G. (2017). Fevipiprant, an oral prostaglandin DP2receptor (CRTh2) antagonist, in allergic asthma uncontrolled on low-dose inhaled corticosteroids. European Respiratory Journal.

[74] Santus P, Radovanovic D (2016). Prostaglandin D2 receptor antagonists in early development as potential therapeutic options for asthma. Expert Opin Investig Drugs.

[75] Kuna P, Bjermer L, Tornling G (2016). Two Phase II randomized trials on the CRTh2 antagonist AZD1981 in adults with asthma. Drug Des Devel Ther.

[76] Erpenbeck VJ, Popov TA, Miller D, Weinstein SF, Spector S, Magnusson B, Osuntokun W, Goldsmith P, Weiss M, Beier J (2016). The oral CRTh2 antagonist QAW039 (fevipiprant): A phase II study in uncontrolled allergic asthma. Pulm Pharmacol Ther.

[77] Hall IP, Fowler AV, Gupta A, Tetzlaff K, Nivens MC, Sarno M, Finnigan HA, Bateman ED, Rand Sutherland E (2015). Efficacy of BI 671800, an oral CRTH2 antagonist, in poorly controlled asthma as sole controller and in the presence of inhaled corticosteroid treatment. Pulm Pharmacol Ther.

[78] Pettipher R, Hunter MG, Perkins CM, Collins LP, Lewis T, Baillet M, Steiner J, Bell J, Payton MA (2014). Heightened response of eosinophilic asthmatic patients to the CRTH2 antagonist OC000459. Allergy.

[79] Fowler A, Koenen R, Hilbert J, Blatchford J, Kappeler D, Benediktus E, Wood C, Gupta A (2017). Safety, Tolerability, Pharmacokinetics, and Pharmacodynamics of the Novel CRTH2 Antagonist BI 1021958 at Single Oral Doses in Healthy Men and Multiple Oral Doses in Men and Women With Well-Controlled Asthma. J Clin Pharmacol.

[80] Gonem S, Berair R, Singapuri A, Hartley R, Laurencin MF, Bacher G, Holzhauer B, Bourne M, Mistry V, Pavord ID (2016). Fevipiprant, a prostaglandin D2 receptor 2 antagonist, in patients with persistent eosinophilic asthma: a single-centre, randomised, double-blind, parallel-group, placebo-controlled trial. Lancet Respir Med.

[81] Fajt ML, Gelhaus SL, Freeman B, Uvalle CE, Trudeau JB, Holguin F, Wenzel SE (2013). Prostaglandin D(2) pathway upregulation: relation to asthma severity, control, and TH2 inflammation. J Allergy Clin Immunol.

[82] Murray JJ, Tonnel AB, Brash AR, Roberts LJ, Gosset P, Workman R, Capron A, Oates JA (1986). Release of prostaglandin D2 into human airways during acute antigen challenge. N Engl J Med.

[83] Wenzel SE, Westcott JY, Larsen GL (1991). Bronchoalveolar lavage fluid mediator levels 5 minutes after allergen challenge in atopic subjects with asthma: relationship to the development of late asthmatic responses. J Allergy Clin Immunol.

[84] Stinson SE, Amrani Y, Brightling CE (2015). D prostanoid receptor 2 (chemoattractant receptor-homologous molecule expressed on TH2 cells) protein expression in asthmatic patients and its effects on bronchial epithelial cells. J Allergy Clin Immunol.

[85] Campos Alberto E, Maclean E, Davidson C, Palikhe NS, Storie J, Tse C, Brenner D, Mayers I, Vliagoftis H, El-Sohemy A, Cameron L (2012). The single nucleotide polymorphism CRTh2 rs533116 is associated with allergic asthma and increased expression of CRTh2. Allergy.

[86] Saunders RM, Kaul H, Berair R, Singapuri A, Chernyasvsky I, Chachi L, Biddle M, Sutcliffe A, Laurencin M, Bacher G (2017). Fevipiprant (QAW039) reduces airway smooth muscle mass in asthma via antagonism of the prostaglandin D2 receptor 2 (DP2). Am J Respir Crit Care Med.

[87] Parameswaran K, Radford K, Fanat A, Stephen J, Bonnans C, Levy BD, Janssen LJ, Cox PG (2007). Modulation of human airway smooth muscle migration by lipid mediators and Th-2 cytokines. Am J Respir Cell Mol Biol.

[88] Shiraishi Y, Asano K, Niimi K, Fukunaga K, Wakaki M, Kagyo J, Takihara T, Ueda S, Nakajima T, Oguma T (2008). Cyclooxygenase-2/prostaglandin D2/CRTH2 pathway mediates double-stranded RNA-induced enhancement of allergic airway inflammation. J Immunol.

[89] Yu M, Levine SJ (2011). Toll-like receptor, RIG-I-like receptors and the NLRP3 inflammasome: key modulators of innate immune responses to double-stranded RNA viruses. Cytokine Growth Factor Rev.

[90] Barnig C., Cernadas M., Dutile S., Liu X., Perrella M. A., Kazani S., Wechsler M. E., Israel E., Levy B. D. (2013). Lipoxin A4 Regulates Natural Killer Cell and Type 2 Innate Lymphoid Cell Activation in Asthma. Science Translational Medicine.

[91] Jackson DJ, Shamji B, Trujillo-Torralbo M-B, Walton RP, Bartlett NW, Edwards MR, Mallia P, Edwards M, Westwick J, Johnston SL (2014). Prostaglandin D2 is induced during rhinovirus-induced asthma exacerbations and related to exacerbation severity in vivo. Am J Respir Crit Care Med.

[92] Sandham D, Asano D, Barker L, Budd D, Erpenbeck V, Knowles I, Mikami T, Profit R, Robb O, Shiraishi Y (2018). Fevipiprant, a potent selective prostaglandin D2 receptor 2 (DP2) antagonist, dose-dependently inhibits pulmonary inflammation in a mouse model of asthma. Am J Respir Crit Care Med.

[93] Russell RJ, Brightling C (2017). Pathogenesis of asthma: implications for precision medicine. Clin Sci (Lond).

[94] Diamant Z, Sidharta PN, Singh D, O'Connor BJ, Zuiker R, Leaker BR, Silkey M, Dingemanse J (2014). Setipiprant, a selective CRTH2 antagonist, reduces allergen-induced airway responses in allergic asthmatics. Clin Exp Allergy.

[95] Santini G, Mores N, Malerba M, Mondino C, Macis G, Montuschi P (2016). Investigational prostaglandin D2 receptor antagonists for airway inflammation. Expert Opin Investig Drugs.

